# Preparation of Well-Dispersed Chitosan/Alginate Hollow Multilayered Microcapsules for Enhanced Cellular Internalization

**DOI:** 10.3390/molecules23030625

**Published:** 2018-03-10

**Authors:** Carla Ribeiro, João Borges, Ana M. S. Costa, Vítor M. Gaspar, Verónica de Zea Bermudez, João F. Mano

**Affiliations:** 1 Department of Chemistry, CICECO—Aveiro Institute of Materials, University of Aveiro, Campus Universitário de Santiago, 3810-193 Aveiro, Portugal; carla_madalena_5@hotmail.com (C.R.); amargaridasc@ua.pt (A.M.S.C.); vm.gaspar@ua.pt (V.M.G.); 2 Department of Chemistry and CQ-VR, University of Trás-os-Montes e Alto Douro, 5001-801 Vila Real, Portugal; vbermude@utad.pt

**Keywords:** chitosan, alginate, marine origin polysaccharides, biocompatible polymers, CaCO_3_ porous microparticles, layer-by-layer assembly, hollow multilayered microcapsules, non-aggregated microcapsules, cellular internalization

## Abstract

Hollow multilayered capsules have shown massive potential for being used in the biomedical and biotechnology fields, in applications such as cellular internalization, intracellular trafficking, drug delivery, or tissue engineering. In particular, hollow microcapsules, developed by resorting to porous calcium carbonate sacrificial templates, natural-origin building blocks and the prominent Layer-by-Layer (LbL) technology, have attracted increasing attention owing to their key features. However, these microcapsules revealed a great tendency to aggregate, which represents a major hurdle when aiming for cellular internalization and intracellular therapeutics delivery. Herein, we report the preparation of well-dispersed polysaccharide-based hollow multilayered microcapsules by combining the LbL technique with an optimized purification process. Cationic chitosan (CHT) and anionic alginate (ALG) were chosen as the marine origin polysaccharides due to their biocompatibility and structural similarity to the extracellular matrices of living tissues. Moreover, the inexpensive and highly versatile LbL technology was used to fabricate core-shell microparticles and hollow multilayered microcapsules, with precise control over their composition and physicochemical properties, by repeating the alternate deposition of both materials. The microcapsules’ synthesis procedure was optimized to extensively reduce their natural aggregation tendency, as shown by the morphological analysis monitored by advanced microscopy techniques. The well-dispersed microcapsules showed an enhanced uptake by fibroblasts, opening new perspectives for cellular internalization.

## 1. Introduction

Over the last two decades, there has been a tremendous interest by the scientific community in the design and fabrication of functional nano- and micro-sized drug carriers with the aim of enhancing the loading, protection, delivery and targeting efficacy of therapeutics and, thus, address numerous biomedical, biotechnological and pharmaceutical applications [1,2,3]. Those nano- and micro-platforms include liposomes [4,5], polymersomes [6,7], micelles [4,8], dendrimers [4,9], inorganic and polymeric particles/capsules [4,10,11,12,13,14,15], and virus- [16,17] or cell-based [18,19,20,21] vehicles.

Among the arsenal of nano- and microcarriers, polymeric capsules, prepared by different engineering strategies [22,23], have emerged as promising drug delivery vehicles for the successful encapsulation, protection and controlled release of bioactive agents at site-specific locations. Since the pioneering work of Möhwald and co-workers in 1998 [13,14,15], the fabrication of polyelectrolyte multilayer capsules has attracted increasing attention in the biological, biotechnological and biomedical fields since their composition, structure, mechanical and physicochemical properties can be precisely and independently tuned at the nanometer scale [24,25,26,27,28]. These capsules are typically engineered by repeating the sequential adsorption of aqueous solutions of oppositely charged polyelectrolytes onto a sacrificial organic or inorganic spherical template using the prominent Layer-by-Layer (LbL) assembly technology, followed by core template dissolution using ethylenediamine-tetraacetic acid (EDTA), organic solvents or acidic solutions. This technology is a simple, inexpensive and reproducible bottom-up surface engineering strategy to fabricate robust, multifunctional multilayered assemblies for addressing a wide variety of applications [29,30]. Moreover, its high versatility and mild processing conditions further enable the modification of any type of template surface, regardless of size and shape, with an unprecedented source of building blocks exhibiting complementary multivalent interactions to add new functionalities, as well as the safe and stable encapsulation of a diverse set of cargo molecules [29,31]. The permeability of the hollow multilayered capsules and the release rate of the encapsulated molecules can be precisely tailored at the nanometer scale by simply changing the number of adsorbed layers (i.e., the thickness of the shell), assembly conditions and the nature of the assembled materials that integrate the multilayer shell [28,29,32,33].

Amongst the diverse set of particles that can be used as templates for the preparation of polyelectrolyte multilayered capsules, porous particles are particularly attractive due to their unique and superior properties. In this context, calcium carbonate (CaCO_3_) is of the utmost interest. This abundant inorganic material undergoes outstanding biomineralization processes that yield beautiful, robust, hierarchically organized, and intricate complex architectures in Nature, such as those produced by coccoliths. Walsh and Mann were the first to produce hollow porous shells of aragonite (an anhydrous CaCO_3_ polymorph less stable than calcite) mimicking the coccospheres of certain marine algae [34]. The use of oil–water–surfactant microemulsions supersaturated with calcium bicarbonate allowed them to produce thin cellular frameworks of either meso- or macroporous aragonite. Hollow spherical shells of the honeycomb architecture were obtained upon incorporation of gold-coated micrometre-sized polystyrene beads acting as template for the microemulsion. Walsh and co-workers reported later an improved method for the synthesis of discrete spherical micrometer-sized particles of vaterite (the most unstable anhydrous CaCO_3_ polymorph) with an elaborate sponge-like microarchitecture [35]. The spheres were produced by nucleation of vaterite at the anionic head groups of sodium dodecylsulfate molecules occurring in the oil/water droplet interface and patterned by CO_2_ microbubbles entrapped within and at the surface of the water droplets. These works clearly demonstrate the versatility of CaCO_3_ as a suitable inorganic template that can be obtained from various techniques and exhibit different sizes and morphologies.

Porous CaCO_3_ particles have received increasing interest as suitable templates for engineering prospective drug delivery vehicles owing to their easy, fast and cost-effective synthesis, large surface area to volume ratio, inherent biocompatibility, high mechanical stability, non-toxicity and mild decomposition conditions [36,37]. In addition, their highly porous structure allows a high loading efficiency of cargo molecules [38,39,40].

In the literature, one can find several works that report the use of CaCO_3_ microcores as templates for the preparation of hollow polyelectrolyte multilayered capsules [38,39,40,41,42]. These capsules have been mostly prepared by assembling, in a LbL fashion, multiple layers of oppositely charged non-degradable synthetic polymers over the sacrificial colloidal core, followed by core decomposition [14,38,39,40]. Besides, multilayered shells comprising solely natural-based polymers [43] and hybrid synthetic/natural materials [44,45,46] have been also templated on CaCO_3_ microcores to prepare hollow multilayered capsules. As it is well-known, the preparation of such capsules using solely natural origin polymers is particularly attractive when aiming at fulfilling biomedical applications, including drug delivery, cellular internalization, or intracellular trafficking, due to their biocompatibility, biodegradability and close structural similarity to the native biological systems. Despite recent advances in the field, to the best of our knowledge, the reported procedures for the synthesis of CaCO_3_-templated natural polymers-based hollow multilayered capsules have been inefficient in preventing their aggregation in solution, which represents a major hurdle when aiming, for instance, cellular internalization [47].

Herein, we report the preparation of well-dispersed marine origin polysaccharide-based hollow multilayered microcapsules through the sequential deposition of oppositely charged chitosan (CHT) and alginate (ALG) layers onto porous CaCO_3_ sacrificial microcores, followed by core template dissolution. CHT and ALG have been chosen as the cationic and anionic biopolymers, respectively, due to their proven biocompatibility, non-cytotoxicity and non-immunogenic properties, being very appealing for biomedical and biotechnological applications. Moreover, both marine origin biopolymers are widely available in the sea, being easily extracted from crustaceans’ shells and brown algae, respectively. The hollow multilayered capsules have been analyzed in terms of net surface charge and morphology by resorting to zeta potential measurements, and advanced microscopy techniques, including scanning electron microscopy (SEM), transmission electron microscopy (TEM), widefield fluorescence (FL) microscopy and confocal laser scanning microscopy (CLSM). Moreover, the effective cellular uptake of the individual micrometer-sized spherical CHT/ALG hollow multilayered microcapsules by fibroblasts was assessed by CLSM.

## 2. Results

**Scheme 1:**
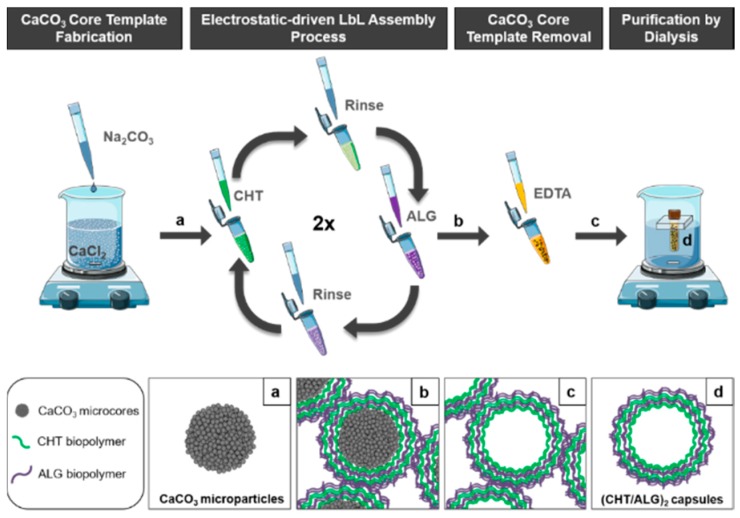
Schematic illustration of the synthesis of (**a**) CaCO_3_ core microparticles, (**b**) core-shell microparticles with two CHT/ALG bilayers, (**c**) (CHT/ALG)_2_-based hollow multilayered microcapsules after CaCO_3_ core template dissolution with EDTA, and (**d**) well-dispersed (CHT/ALG)_2_-based hollow multilayered microcapsules after purification by dialysis.

In this work, porous CaCO_3_ microparticles were synthesized at room temperature by co-precipitation of equal volumes of 0.33 M Na_2_CO_3_ and 0.33 M CaCl_2_ aqueous solutions under vigorous stirring followed by washing and centrifugation steps. These particles were further employed as sacrificial templates for the preparation of well-dispersed polysaccharide-based hollow multilayered microcapsules with enhanced potential for cellular uptake. As depicted in the Scheme 1, the polysaccharide-based hollow multilayered microcapsules were obtained by resorting to marine origin polysaccharides, the LbL assembly technology, core template dissolution with EDTA calcium-chelating agent, and purification steps.

### 2.1. Morphological Characterization of Porous CaCO_3_ Microparticles

**Figure 1:**
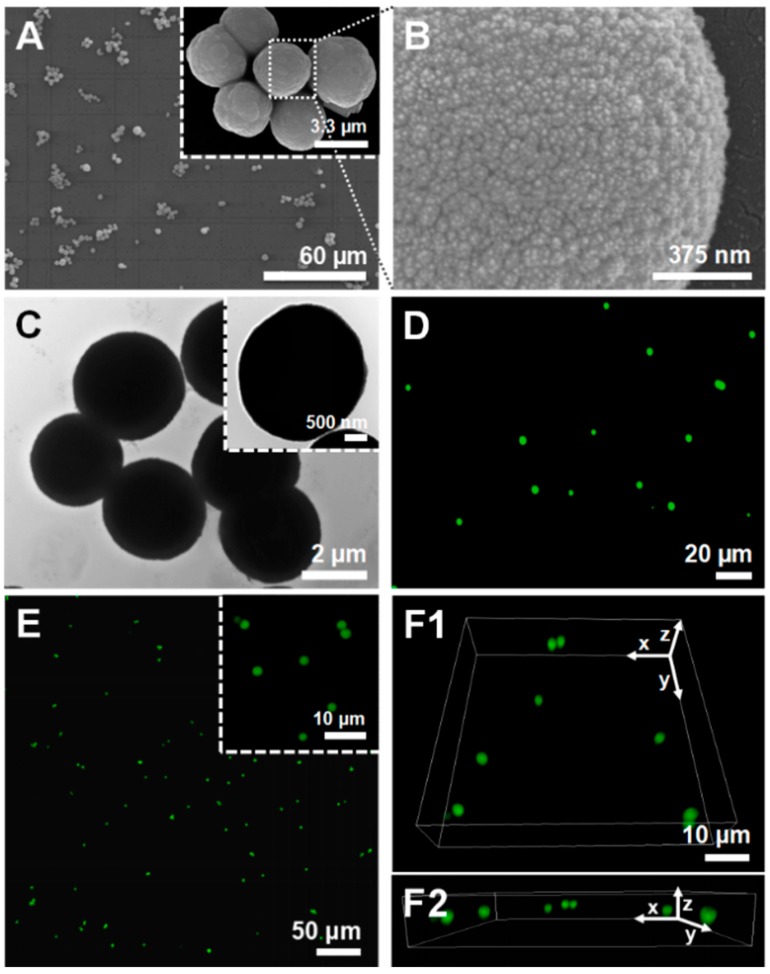
(**A**,**B**) SEM, (**C**) TEM, (**D**) FL microscopy and (**E**,**F**) CLSM micrographs of the uncoated CaCO_3_ microparticles prepared at room temperature. (**B**) The SEM micrograph shows the high surface roughness and the porous structure of the microparticles. (**D**) FL microscopy and (**E**,**F**) 3D reconstructed CLSM micrographs were acquired by loading the CaCO_3_ core microparticles with FITC (green channel). **F1** and **F2** correspond to the upper (xy) and lateral (xz) views, respectively.

The surface morphology of the uncoated and unloaded CaCO_3_ microparticles was investigated by SEM and TEM. Figure 1A–C shows SEM and high-resolution TEM images of the as-prepared CaCO_3_ microcores, revealing the preparation of homogeneous spherical microparticles with a high surface roughness and porous structure. The average diameter of the core microparticles, estimated from SEM and TEM images, was determined to be 3.5 ± 1.4 µm. Although the average particle size decreased slightly when compared with the size reported in the literature for CaCO_3_ microparticles prepared following the same experimental conditions (~4–6 µm) [38,39,40], one should bear in mind that the particles were air-dried upon sample preparation for SEM and TEM analysis, which could have contributed for the reduction in size. Moreover, FITC-loaded CaCO_3_ microcores were also prepared to assess their average diameter and possible aggregation in solution by FL microscopy (Figure 1D) and CLSM (Figure 1E,F). Figure 1D–F reveals the preparation of well-dispersed and size-uniform spherical microspheres. The CLSM micrographs not only corroborated the FL microscopy images but also enabled the acquisition of high resolution images with multiple focal planes, providing three-dimensional (3D) data (Figure 1E,F). In addition, the average particle size was determined to be 4.2 ± 1.3 µm, which corroborates the sizes reported in previous studies [38,39,40].

### 2.2. Physicochemical and Morphological Characterization of Core-Shell CaCO_3_ Microparticles Coated with CHT/ALG Multilayered Shells

Following their synthesis, porous CaCO_3_ microspheres were used as templates for the fabrication of core-shell microparticles with polysaccharide multilayers. The particle shell was prepared by resorting to multilayers of oppositely charged biocompatible CHT and ALG biopolymers assembled on particles’ surface via electrostatic-driven LbL assembly approach.

#### 2.2.1. Zeta (ζ)-Potential Measurements

The stability of CaCO_3_ microparticles and the successful interaction and assembly of both biopolymers on the microparticles was firstly monitored by zeta (ζ)-potential measurements. However, before carrying out the LbL assembly process to assemble the core-shell polysaccharide-coated microparticles, the net electrical charge of the uncoated CaCO_3_ microparticles suspension, as well as those individual CHT and ALG bulk aqueous solutions, were evaluated to assess the possible electrostatic attractive interaction between the biopolymers.

**Figure 2:**
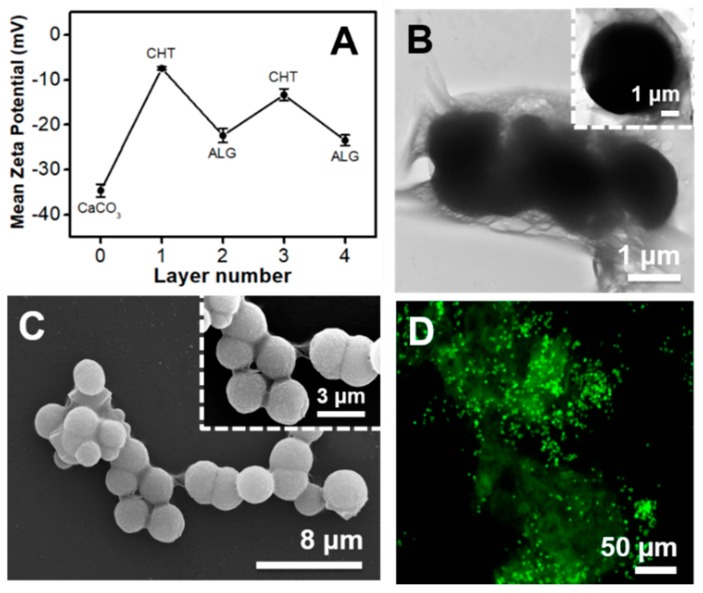
(**A**) Mean ζ-potential as a function of the polysaccharide layer number for the CaCO_3_ microparticles coated with alternating CHT and ALG layers. The odd layer numbers correspond to the CHT deposition and the even layer numbers to the ALG deposition. (**B**) TEM, (**C**) SEM and (**D**) FL microscopy micrographs of the core-shell CaCO_3_ microparticles after the deposition of two CHT/ALG bilayers. The insets in (**B**) and (**C**) show a higher magnification of the TEM and SEM images, respectively. The inset in (**C**) also shows the high surface roughness of the core-shell microparticles.

As it is well-known, CaCO_3_ microparticles in the vaterite form are not stable in aqueous solution and, thus, they recrystallize in water within a few hours into the most stable non-porous calcite phase at ambient conditions [38]. This means that the spherical and porous CaCO_3_ microparticles, which are very attractive for biomedical and biotechnological applications, easily transform into a non-porous rhombohedral crystal shape material once exposed to water. To circumvent this hurdle, immediately after the synthesis procedure, the ζ-potential of the microparticle suspension was measured and the particles were immediately dried at 70 °C for 1 h and kept as a powder until further use. As shown in Figure 2A, the CaCO_3_ microparticles showed a high negative surface charge of −34.6 ± 1.4 mV, which prevented their aggregation in aqueous solution. Then, before proceeding for the preparation of the core-shell polysaccharide-coated microparticles, the electrical charges of the bulk CHT and ALG aqueous solutions at the working pH of 5.5 were measured. The freshly prepared CHT and ALG aqueous solutions exhibited ζ-potentials of +21.5 ± 0.6 mV and −31.2 ± 2.0 mV, thus confirming the cationic (pH < p*K*_a_ ~6–6.5) [48] and anionic (pH > p*K*_a_ ~3.38 or 3.65 for mannuronic or guluronic acid residues, respectively) [49,50] nature of the CHT and ALG biopolymer solutions, respectively. Based on the gathered values, we have assessed the stability and possible step-by-step build-up of a two CHT/ALG bilayer shell on the CaCO_3_ microparticles’ surface by employing the electrostatic-driven LbL assembly interaction between oppositely charged materials. Figure 2A shows the ζ-potential measurements as a function of the biocompatible CHT/ALG layer number deposited on the CaCO_3_ core templates. The LbL assembly process initiated with the deposition of a first CHT layer owing to the negative net surface charge shown by the CaCO_3_ microparticles. As seen in Figure 2A, the adsorption of the first CHT layer (1st odd layer) on the particles surface resulted in a noticeable increase in the ζ-potential value (−7.5 ± 0.4 mV). However, it did not enable a complete charge reversal, as the ζ-potential value remained still below zero. This behavior could be due to the relatively low amount of protonated CHT primary amine groups at the working pH of 5.5 when compared with the electrical charge measured for the uncoated microparticles, as well as to a moderate change in the pH while coating the particles with a CHT layer [51]. The deposition of the first ALG layer (1st even layer), i.e., second biopolymer layer, on the CHT-coated CaCO_3_ particles, resulted in a significant decrease in the ζ-potential, which turned more negative (−22.4 ± 1.6 mV), thus supporting the deposition of the negatively charged ALG layer on the CHT-coated particles. The subsequent deposition of the third (2nd odd layer) and fourth (2nd even layer) biopolymer layers on the CHT/ALG-coated particles, respectively CHT (−13.3 ± 1.3 mV) and ALG (−23.4 ± 1.2 mV) layers, followed the same trend. A similar behavior was previously reported by Möhwald and co-workers for the deposition of the same CHT/ALG multilayers on poly(dl-lactic acid) (PDLLA) microparticles [51]. The authors postulated that the negative ζ-potential values measured after the deposition of the CHT layers on the PDLLA template could be due to a pH shift during the adsorption of the CHT layers. A different phenomenon has been reported in the literature for the deposition of synthetic polyelectrolytes, including the widely explored non-biocompatible poly(sodium 4-styrenesulfonate) (PSS)/poly(allylamine hydrochloride (PAH) polyelectrolyte layer pair. The sequential deposition of negatively charged PSS and positively charged PAH layers on several particle templates led to a stable switching of the ζ-potential values from negative to positive values after the deposition of each polymer layer, respectively [14,15,21,38,39,40]. Although we have not seen a typical net surface charge reversal between positive and negative values after the deposition of the positively charged CHT and negatively charged ALG layers on the CaCO_3_ microparticles, respectively, which would indicate the successful step-by-step build-up of CHT/ALG bilayers, the fabrication of core-shell (CHT/ALG)_2_-coated microparticles was obvious from advanced microscopy techniques, including TEM, SEM and FL microscopy images, as shown in Figure 2B–D.

#### 2.2.2. Morphological Characterization

The inspection of the surface morphology of the core-shell particle structure was assessed by SEM (Figure 2C), which revealed the porous structure and the increase in the surface roughness when compared with the uncoated microparticles (Figure 1). Such increase in the surface roughness was the result of the formation of a regular CHT/ALG multilayered coating on the CaCO_3_ microparticles surface. Moreover, the formation of particle aggregates is clearly perceived. Apart from these aggregates and the higher surface roughness, the multilayered coating did not induce significant changes in the surface morphology of the particles. In addition, particle coalescence is also observed at the surface where the core-shell particles are in contact (Figure 2B,C). This phenomenon could be ascribed to the aggregation of the biopolymer multilayered coating on each particle upon drying and to the lack of enough electrostatic repulsion between the microparticles [52], corroborating the ζ-potential data (Figure 2A). To assess the influence of sample drying, required for SEM and TEM analysis, on the formation of particle aggregates, FL microscopy images were acquired for the (FITC-CHT/ALG)_2_-coated CaCO_3_ microparticles suspension (Figure 2D). As shown in Figure 2D, the core-shell particles demonstrated a high degree of aggregation in solution, thus revealing that the particle aggregates, observed upon drying the core-shell particles for the TEM (Figure 2B) and SEM (Figure 2C) analysis, are mostly due to preformed aggregates in solution. In addition, it is evident that the (CHT/ALG)_2_-coated microparticles (Figure 2) showed a high degree of aggregation when compared with the uncoated ones (Figure 1), confirming the presence of the CHT/ALG multilayered coating. This is particularly visible through the comparison of the FL microscopy images of the coated- (Figure 2D) and uncoated (Figure 1D) CaCO_3_ core microparticles.

### 2.3. Physicochemical and Morphological Characterization of CHT/ALG Hollow Multilayered Microcapsules Templated on CaCO_3_ Microparticles

**Figure 3:**
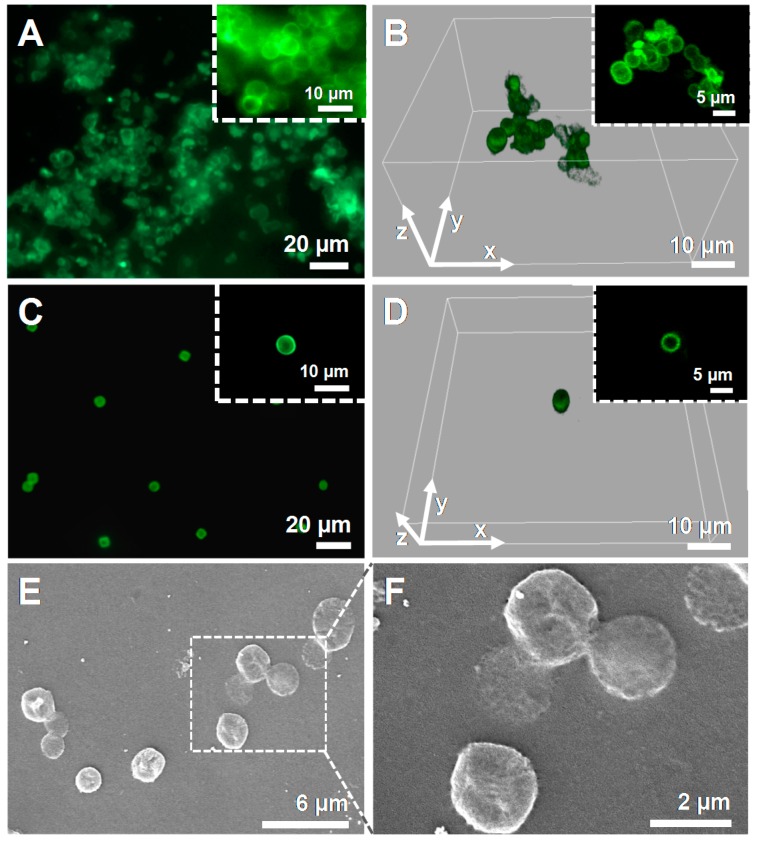
(**A**,**C**) FL microscopy and (**B**,**D**) CLSM micrographs of (CHT/ALG)_2_-based hollow multilayered microcapsules before (**A**,**B**) and after (**C**,**D**) the dialysis purification step. Green channel: FITC-labeled CHT. The insets in **B**,**D** correspond to the middle plane of the microcapsules. (**E**,**F**) SEM micrographs of the (CHT/ALG)_2_-based hollow multilayered microcapsules after dialysis, showing folds and creases in their structure due to the drying procedure.

The core-shell porous CaCO_3_ microparticles with two CHT/ALG bilayers were further employed for the fabrication of well-dispersed biocompatible CHT/ALG hollow multilayered microcapsules after the dissolution of the core template with EDTA. Figure 3A,B shows the FL microscopy and CLSM images of the hollow multilayered microcapsules comprising two CHT/ALG bilayers, respectively, revealing capsules with an average diameter of 4.8 ± 0.9 µm. Indeed, it is clear that the hollow microcapsules maintained the core spherical shape after the decomposition of the core template, and that the capsules aggregated in solution. A similar behavior was seen for the (CHT/ALG)_2_-coated CaCO_3_ microparticles (Figure 2D). Moreover, some particle coalescence is also clearly observed at the surface where the core-shell particles are in contact (see insets on Figure 3A,B). As abovementioned, the aggregation of the biopolymer coating on each particle could be due to the drying procedure, as well as to the lack of enough electrostatic repulsion between the particles owing to their micrometer-size [52]. Additionally, the cross-linking of the ALG layers by divalent calcium ions, released from the particle core to the surrounding media upon core template dissolution, could also contribute to the aggregation of the microcapsules [10,49].

The fabrication of well-dispersed hollow multilayered capsules is particularly desired when aiming for cellular internalization [53,54]. Based on the gathered FL and CLSM data, one could hypothesize that the aggregation observed on the hollow multilayered microcapsules would not enable their uptake by cells, whose size is in the order of a few tens of micrometers. Hence, we have looked at a way to prepare individual and well-dispersed CHT/ALG hollow multilayered microcapsules, with individual size of a few micrometers, to allow their uptake by cells. For that, after the preparation of the CHT/ALG hollow multilayered microcapsules, the capsules were dialyzed against an acetate buffer solution at pH 5.5 for 24 h to remove unbound materials. Figure 3C–F depicts the FL microscopy (Figure 3C), CLSM (Figure 3D) and SEM (Figure 3E,F) micrographs of the (CHT/ALG)_2_-based hollow multilayered microcapsules after being submitted to the dialysis purification step. As shown by the Figure 3C,D the dialysis enabled the preparation of individual, well-dispersed spherical microcapsules with an average diameter of 4.5 ± 0.8 µm, having immense potential for cellular uptake. Their surface morphology was further analyzed by SEM. Figure 3E,F reveals that the CHT/ALG multilayered shells are continuous. Furthermore, capsules with folds and creases are noticed, which arise from the drying procedure upon capsules preparation for SEM analysis, leading to their collapse.

### 2.4. In Vitro Cellular Uptake of CHT/ALG Hollow Multilayered Microcapsules Templated on CaCO_3_ Microparticles

**Figure 4:**
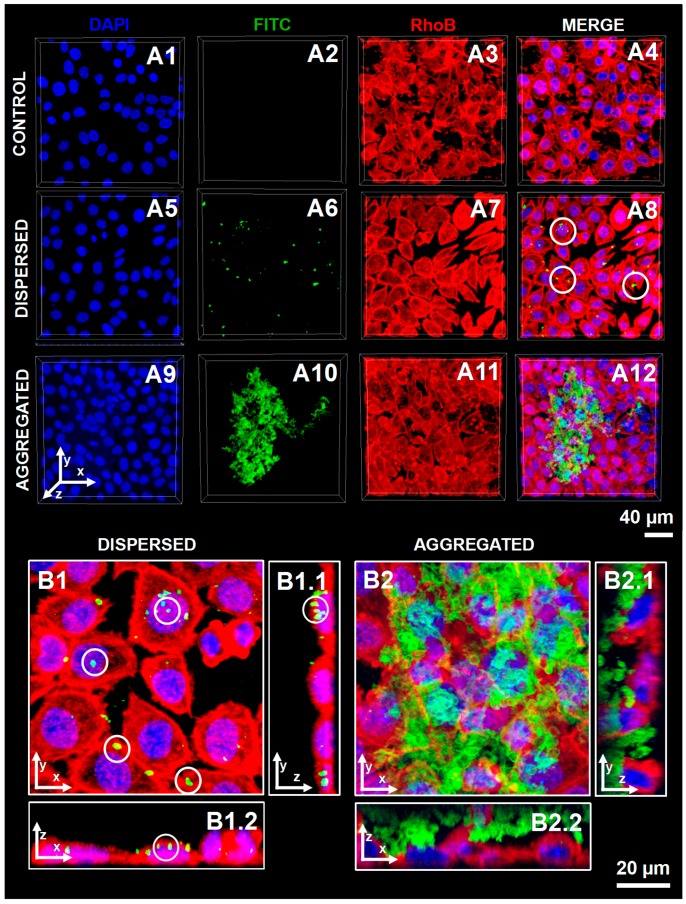
CLSM images of L929 cells upon contacting with well-dispersed or aggregated (FITC-CHT/ALG)_2_ hollow multilayered microcapsules. (**A1**–**A4**) Cells that were not in contact with microcapsules were used as controls. Dispersed (FITC-CHT/ALG)_2_ hollow multilayered microcapsules were internalized by cells (**A5**–**A8**). Contrarily, aggregated capsules were not incorporated and remained adsorbed on the cell surface (**A9**–**A12**). A zoomed-in region of (**A8**,**A12**) micrographs was done to distinguish the internalized dispersed capsules (**B1**–**B1.2**) from the ones adsorbed at the cell surface due to their higher aggregation degree (**B2**–**B2.2**). (**B1**,**B2**) correspond to 3D reconstructed z-stack micrographs as seen from top (view of the xy plane). **B1.1**–**B2.1** and **B1.2**–**B2.2** correspond to orthogonal projection images (view of the yz plane and view of the xz plane, respectively). Blue channel: DAPI nuclear probe; Red channel: rhodamine-labeled phalloidin; Green channel: (FITC-CHT/ALG)_2_-based hollow multilayered microcapsules. White circles indicate some examples of microcapsules internalized by L929 cells.

The cellular uptake of polysaccharide-based hollow microcapsules was investigated by incubating cultured L929 fibroblasts with either dispersed or aggregated capsules comprising FITC-CHT/ALG multilayers. Figure 4 summarizes the data obtained by CSLM, where cells nucleus and their cytoskeleton were stained with blue-fluorescent DAPI and red-fluorescent RhoB-phalloidin, respectively. Regarding the L929 cell morphology, no significant differences were observed after the contact between these cells and the different multilayered microcapsules formulations, since the cells exhibit their typical fibroblast-like shape (Figure 4A1 vs. A5 and A9 and Figure 4A3 vs. A7 and A11). These results were corroborated by previous studies reporting the non-cytotoxic nature of all the materials used in this work, namely the CaCO_3_ microcores [55], and CHT [48] and ALG [49] biopolymers, highlighting the suitability of these particles as cell internalization devices. The comparison of Figure 4A8 and A12 demonstrates that, after a 24 h incubation period, aggregated hollow multilayered microcapsules were not internalized by cells, remaining adsorbed on the cell membrane. In contrast, well-dispersed (FITC-CHT/ALG)_2_-based hollow microcapsules were incorporated within L929 cells, as easily seen on the 3D-reconstructed CSLM images (see Figure 4B). Indeed, the internalized microcapsules, marked with a white circle, appear in yellow as an overlap between the green and red fluorescence channels. 

Previous studies demonstrated that cells can incorporate capsules with a few micrometers [53], without significant changes on the cell height. This engulfment process was attributed to a non-specific interaction between the particle and the cell membrane, owing to the lack of cell-specific ligands on the particle surface [54]. It is well-known that particles’ charge has a significant role on their cellular uptake since the cell membrane has an overall net negative charge with some positively charged domains. Several studies of particles uptake suggest a faster cellular uptake rate of charged particles when comparing with uncharged ones [56]. Moreover, positively charged capsules have shown to be more easily internalized than negatively charged ones [57,58]. This phenomenon has been ascribed to the enhanced electrostatic-driven interaction between the positively charged particles and the overall negatively charged cell membrane [57]. Although the produced hollow multilayered microcapsules ended with a negatively charged layer, they could be engulfed due to a shielding of the microcapsule surface charge by non-specifically adsorbed molecules from the serum supplemented cell culture medium, in a way that the previous differences in the microcapsules surface charge vanished [57].

Interestingly, the internalized microcapsules showed a smaller size and a deformed structure when compared to their non-internalized counterparts. This phenomenon was previously reported in the literature, being ascribed to the squeezing of the flexible nanometer-scale multilayered shell upon cellular uptake, as well as to the more acidic environment to which capsules might be exposed during intracellular trafficking (Figure 4B1 vs. B2) [59,60].

In conclusion, the well-dispersed hollow multilayered microcapsules could be used as suitable carrier vehicles of various bioactive molecules into human cells for pharmaceutical, biomedical and biotechnological applications, a topic which is currently under investigation in our laboratory. 

## 3. Materials and Methods

### 3.1. Materials

Anhydrous calcium chloride (CaCl_2_) and sodium chloride (NaCl) were purchased from PanReac AppliChem (Darmstadt, Germany) and glacial acetic acid (CH_3_COOH) was purchased from VWR International (West Chester, PA, USA). Fluorescein 5(6)-isothiocyanate (FITC), ethylenediaminetetraacetic acid (EDTA), sodium carbonate anhydrous (Na_2_CO_3_), sodium acetate trihydrate (CH_3_COONa∙3H_2_O, ≥99%), sodium hydroxide (NaOH), hydrochloric acid (HCl, 37%), sodium alginate derived from brown algae (ALG, *M*_w_ ~538 kDa, viscosity ~250 cP), Dulbecco’s Modified Eagle’s Medium (DMEM) low glucose, formalin, Triton™ X-100 BioXtra, sodium bicarbonate powder suitable for cell culture and antibiotic-antimycotic solution (100×, with 10,000 units penicillin, 10 mg streptomycin and 25 μg amphotericin B per mL) were purchased from Sigma-Aldrich (St. Louis, MO, USA). Rhodamine phalloidin, 4′,6-diamidino-2-phenylindole (DAPI), trypLE™ express solution and fetal bovine serum (FBS) were acquired from Alfagene (Lisbon, Portugal). Dulbecco’s phosphate-buffered saline (DPBS) was obtained from Thermo Fisher Scientific (Fair Lawn, NJ, USA). An immortalized mouse lung fibroblast cell line (L929, European Collection of Authenticated Cell Cultures) was supplied by Sigma-Aldrich (St. Louis, MO, USA). Cellulose ester Float-A-Lyzer^®^ (Los Angeles, CA, USA) dialysis membranes (MWCO = 3.5–5 kDa) and Standard RC tubing (MWCO = 6–8 kDa) were acquired from Spectrum Laboratories Inc. (Los Angeles, CA, USA) and used for purification. Medium molecular weight chitosan (CHT, *M*_w_ ~190–310 kDa, 82.6% degree of deacetylation, viscosity 200–800 cP) was purchased from Sigma-Aldrich (St. Louis, MO, USA) and purified by a series of filtration and precipitation steps in water and ethanol, followed by freeze-drying, as described elsewhere [61]. All the other reagents were used as received without further purification. The CHT and ALG solutions, at a concentration of 2 mg/mL, were prepared in 0.5 M NaCl aqueous solution at pH 5.5. The pH of the polysaccharides and stock solutions were adjusted to pH 5.5 using 1 M HCl and/or 1 M NaOH aqueous solutions. Deionized (DI) water was used throughout the experiments.

### 3.2. Synthesis of Fluorescein Isothiocyanate-Labeled Chitosan Biopolymer (FITC-CHT)

The synthesis of FITC-labeled CHT was carried out following a previous report [62]. Briefly, purified CHT (200 mg) was dissolved overnight in 0.1 M acetic acid (20 mL). Then, anhydrous methanol (20 mL) was added to the CHT solution with continuous stirring for 3 h. Afterwards, FITC (10 mL) dissolved in anhydrous methanol at 2 mg/mL was added to the CHT solution. The reaction between the isothiocyanate group of FITC and the primary amino group of the d-glucosamine residue proceeded for 4 h in the dark at room temperature. Then, the FITC-labeled CHT (FITC-CHT) was precipitated in 0.2 M NaOH and washed with 70% methanol until the supernatant was free of fluorescence. FITC-CHT was dissolved in 0.1 M acetic acid and dialyzed against DI water for 7 days (Standard RC tubing with a MWCO = 6–8 kDa, Spectrum Laboratories Inc., Los Angeles, CA, USA). The whole reaction and dialysis purification process were protected from light. Finally, FITC-CHT was freeze-dried and stored at 4 °C until further use.

### 3.3. Synthesis of Unloaded and FITC-Loaded CaCO_3_ Microparticles

Porous spherical CaCO_3_ microparticles with a narrow size distribution ranging from 4 to 6 µm were prepared as described elsewhere [38]. Briefly, 0.33 M Na_2_CO_3_ aqueous solution (2 mL) was rapidly poured into an equal volume of 0.33 M CaCl_2_ aqueous solution at room temperature, and the mixture was vigorously stirred with a magnetic stirrer for 30 s at 600 rpm. After 30 s the magnetic stirrer was stopped and the suspension of newly synthesized CaCO_3_ microparticles left to react and precipitate for 15 min, resulting in spherical microspheres with an average diameter ranging from 4–6 µm. The microparticles were then washed three times with DI water by centrifugation at 500× *g* for 5 min. Then, the porous and spherical CaCO_3_ vaterite microparticles were immediately dried at 70 °C for 1 h, to avoid their recrystallization into the most thermodynamically stable non-porous rhombohedral calcite form, and kept as a powder until further use.

The synthesis of FITC-loaded CaCO_3_ microparticles followed the same synthesis procedure as described for the preparation of unloaded CaCO_3_ microparticles with a slight modification that comprised the addition of 4 mg of FITC to a 0.33 M CaCl_2_ aqueous solution (2 mL) prior to the preparation of the CaCO_3_ microparticles.

### 3.4. Fabrication of CaCO_3_-Templated Polysaccharide-Based Hollow Multilayered Capsules

Polysaccharide-based hollow multilayered microcapsules were fabricated at room temperature via LbL assembly approach using CaCO_3_ microparticles as sacrificial templates. The sacrificial templates were sequentially immersed in CHT and ALG aqueous solutions (2 mg/mL in 0.5 M NaCl at pH 5.5) for 15 min each with mild agitation, followed by centrifugation at 1000× *g* for 5 min. After the deposition of each biopolymer layer, the coated-microparticles were washed three times with 0.01 M NaCl aqueous solution at pH 5.5 for 5 min each, under gentle shaking, and centrifuged after each washing step at 1000× *g* for 5 min. The washing steps were applied to remove weakly adsorbed biopolymer molecules. The LbL assembly process was repeated four times until reaching two CHT/ALG bilayers. Then, the polysaccharide multilayer-coated CaCO_3_ microparticles were resuspended in a 0.2 M EDTA aqueous solution at pH 5.5 to dissolve the core and form the polysaccharide-based hollow multilayered capsules. Briefly, the LbL coated-microparticles were incubated in the EDTA solution for 1 h with gentle shaking, followed by centrifugation at 5000× *g* for 10 min. Then, the supernatant was removed and the microcapsules were incubated in a fresh EDTA aqueous solution (pH 5.5) overnight with mild agitation. The suspension of the microcapsules was washed three times with 0.1 M acetate buffer solution at pH 5.5 for 5 min each with gentle shaking, centrifuged after each washing step at 5000× *g* for 10 min, and dialyzed against 0.1 M acetate buffer solution at pH 5.5 for 24 h (Float-A-Lyzer dialysis membrane with a MWCO = 3.5–5.0 kDa, Spectrum Laboratories) to remove weakly adsorbed molecules (see Scheme 1). Finally, the polysaccharide-based hollow multilayered microcapsules were stored in water at 4 °C until further use. The same procedure was followed to fabricate (FITC-CHT/ALG)_2_-based hollow multilayered microcapsules templated on CaCO_3_ sacrificial microparticles for FL microscopy and CLSM analysis.

### 3.5. Zeta (ζ)-Potential Measurements

Prior to the preparation of the polysaccharide multilayers-coated CaCO_3_ sacrificial microparticles, the electrophoretic mobility of the individual CHT and ALG aqueous solutions (2 mg/mL, 0.5 M NaCl at pH 5.5) were investigated by zeta (ζ)-potential measurements, to assess their possible interaction via electrostatic interactions. Then, the successful adsorption of multiple layers of CHT and ALG on the core microparticles was assessed after the deposition of each biopolymer layer on the sacrificial microparticles, following the LbL assembly process. After the deposition of each biopolymer layer, the coated CaCO_3_ microparticles were redispersed in 0.01 M NaCl aqueous solution at pH 5.5 prior to the measurement. The zeta (ζ)-potentials of the individual CHT and ALG solutions, as well as (CHT/ALG)_2_-coated CaCO_3_ microcores were measured at 25 °C based on the electrophoretic mobility under an electric field using a Zetasizer Nano-ZS (Malvern Instruments Ltd., Royston, Hertfordshire, UK). The electrophoretic mobility (*u*) was then converted into a zeta (ζ)-potential value using the Smoluchowski relation (ζ = *uη*/*ε*, where *η* and *ε* are the viscosity and permittivity of the solution, respectively) [63]. The measurements were performed in triplicate and averaged for each sample.

### 3.6. Scanning Electron Microscopy (SEM)

Before SEM analysis, glass coverslips were fixed to aluminum stubs by double-sided carbon conductive adhesive tape for electrical contact purposes. Then, a drop of a suspension of uncoated-, (CHT/ALG)_2_-coated CaCO_3_ microparticles or (CHT/ALG)_2_-based hollow multilayered microcapsules was applied to a coverslip and dried overnight at room temperature. All samples were sputtered with gold before being analyzed on a field emission gun scanning electron microscope (FEG SEM Hitachi S4100, Tokyo, Japan) operated in the secondary electrons mode at an acceleration voltage of 25 kV.

### 3.7. Transmission Electron Microscopy (TEM)

TEM analysis was performed on a H9000 instrument (Hitachi, Tokyo, Japan) operated at an acceleration voltage of 300 kV. After the preparation of the uncoated-, (CHT/ALG)_2_-coated CaCO_3_ microparticles, and (CHT/ALG)_2_-based hollow multilayered microcapsules, a drop of each sample suspension was drop casted onto a carbon film-coated copper TEM grid (CF400-Cu—carbon film 400 square mesh copper grid) and left to dry overnight at room temperature before analysis.

### 3.8. Fluorescence (FL) Microscopy

FL microscopy micrographs of uncoated FITC-loaded CaCO_3_ microparticles, (FITC-CHT/ALG)_2_-coated CaCO_3_ microparticles, and (FITC-CHT/ALG)_2_-based hollow multilayered microcapsules before and after dialysis were acquired in an upright motorized widefield microscope (Axio Imager M2, Carl Zeiss, Jena, Germany) equipped with 200 W HXP lamp and a 3.0 Mpix monochromatic camera (Axiocam 105 mono) (Carl Zeiss, Jena, Germany). Micrographs were acquired with an EC plan neofluar 40×/0.75 Ph 2 objective using the eGFP filter block (Carl Zeiss, Jena, Germany) (excitation BP470/40, emission BP525/50). All images were processed in Zeiss Zen Blue software (2017 version) (Carl Zeiss, Jena, Germany).

### 3.9. In Vitro Cellular Uptake Assays

L929 cells were cultured in complete DMEM low glucose medium supplemented with 3.7 g/L sodium bicarbonate, 10% (*v*/*v*) FBS and 1% penicillin-streptomycin at pH 7.4. Cells were grown in 75 cm^2^ tissue culture flasks and incubated at 37 °C in a humidified air atmosphere with 5% CO_2_. The culture medium was exchanged every 3 days. At 90% of confluence, cells grown on tissue culture flasks were washed with DPBS and then harvested using trypLE™ express solution for 5 min at 37 °C in a 5% CO_2_ atmosphere. To inactivate the trypLE™ express effect, cell culture medium was added. The cells were then centrifuged at 300× *g* for 5 min and the medium was decanted. Prior to cell seeding, (FITC-CHT/ALG)_2_-based hollow multilayered microcapsules obtained either before or after dialysis were washed three times with DPBS and once with fresh DMEM culture medium. Between the washing steps, capsules were centrifuged at 5000× *g* for 10 min to separate them from the washing solution. For the uptake studies, cells were seeded for 24 h on 8-well μ-Slide flat bottom imaging plates (Ibidi GmbH, Martinsried, Germany) at a density of 2 × 10^4^ cells per well and with 200 μL of fresh DMEM culture medium. After a 24 h incubation period, cells were incubated with (FITC-CHT/ALG)_2_-based hollow multilayered microcapsules for another 24 h at 37 °C, in a humidified atmosphere containing 5% CO_2_. In addition, L929 cells that were not in contact with microcapsules were used as controls.

### 3.10. Confocal Laser Scanning Microscopy (CLSM)

In vitro uptake of the produced (FITC-CHT/ALG)_2_-based hollow multilayered microcapsules, before and after dialysis, was assessed by CLSM. Briefly, after incubating for 24 h with (FITC-CHT/ALG)_2_-based hollow multilayered microcapsules, cells were fixed and their cell compartments stained using DAPI, to stain the nucleus, and rhodamine-phalloidin to stain F-actin from the cell cytoskeleton. Firstly, samples were gently washed with sterile DPBS and fixed with 200 μL of a 10% (*v*/*v*) formalin in DPBS solution for 30 min at room temperature. The fixed samples were then rinsed with DPBS, permeabilized with 200 μL of 0.2% (*v*/*v*) Triton X-100 in DPBS solution for 10 min and blocked with 200 μL of a 3% (*w*/*v*) BSA in DPBS solution for 30 min. After blocking, cells were incubated for 45 min with rhodamine-phalloidin followed by DAPI for 15 min. Afterwards, samples were washed three times with DPBS and visualized in a LSM 510 Meta confocal microscope (Carl Zeiss, Jena, Germany), using a 63×/1.25 Plan-Apochromat (Carl Zeiss, Jena, Germany) oil objective. All images were recorded as z-stacks and 3D reconstructed using the Zeiss Zen Blue software (2010 version).

### 3.11. Statistical Analysis

All experiments were performed in triplicate (*n* = 3) and the results presented as mean ± standard deviation.

## 4. Conclusions

In this work, individual and well-dispersed CaCO_3_-templated marine origin polysaccharide-based hollow multilayered microcapsules were successfully prepared by exploiting the electrostatic-driven LbL interaction between positively and negatively charged CHT and ALG layers, respectively, followed by the decomposition of the core template with EDTA. The alternate deposition of biocompatible CHT and ALG polymers was monitored by ζ-potential measurements. Moreover, their morphological properties were assessed by SEM, TEM, FL microscopy, and CLSM, showing the fabrication of non-aggregated spherical microcapsules with an average diameter of 5 µm. An enhanced uptake of the well-dispersed CHT/ALG hollow multilayered microcapsules by fibroblast cells was observed by CLSM, which proved their suitability for cellular internalization. The developed biocompatible hollow multilayered microcapsules hold great promise for being used as micro-reservoirs for the successful encapsulation, protection and on-demand controlled release of bioactive molecules, opening new avenues in drug/gene delivery, intracellular trafficking and tissue engineering strategies.
